# Effects of LED Versus Fluorescent Task Lighting on Sleep Quality and Daytime Function in Windowless Office Environments

**DOI:** 10.3390/ijerph22091436

**Published:** 2025-09-16

**Authors:** Juntae Jake Son

**Affiliations:** Department of Construction Management and Interior Design, College of Architecture and Planning, Ball State University, 2000 W. University Ave., Muncie, IN 47306, USA; jjson@bsu.edu; Tel.:+1-765-285-1433

**Keywords:** artificial lighting, LED, fluorescent lighting, circadian rhythm, sleep duration, human well-being

## Abstract

This study investigated the effects of LED versus fluorescent task lighting on sleep quality and daytime functioning among office workers in a windowless environment. Using a within-subjects crossover design with 32 full-time employees, participants were exposed to both 4000 K LED and 4100 K fluorescent lighting conditions over two one-week periods. Subjective sleep quality and alertness were assessed through the Pittsburgh Sleep Quality Index (PSQI) and daily sleep diaries. Results indicated significantly better global sleep quality, improved subjective sleep assessments, and reduced daytime dysfunction under LED lighting conditions. While sleep duration did not significantly differ, a positive trend was observed favoring the LED condition. These outcomes are likely due to differences in spectral power distribution between the two light sources, particularly the continuous, blue-enriched spectrum of the LED lamp, which supports circadian regulation. The findings suggest that biologically supportive lighting—such as continuous-spectrum LEDs—can positively impact sleep and daytime performance, even in the absence of natural daylight. This research contributes to the growing field of circadian lighting and offers practical implications for architects, designers, and workplace managers aiming to enhance employee well-being and productivity in enclosed office environments.

## 1. Introduction

In modern interior work environments, natural lighting access is frequently limited or absent [1,2,3,4,5]. Numerous office environments, such as those within basements, interior buildings, or converted temporary facilities, lack windows, posing challenges not only for visual comfort and energy efficiency but also for health [5,6,7,8,9,10]. Specifically, insufficient daylight can cause disturbances of the sleep-wake cycle and decreased alertness during the day [1,2,3].

One of the body’s main biological systems that is impacted by exposure to lights is the circadian rhythm, our built-in 24 h body clock that regulates sleep, hormone production, and general wakefulness [11,12,13,14,15]. Light is also the most powerful environmental signal that adjusts that intrinsic system, through special non-visual eye receptors. These evoke signals that are transmitted to the brain hypothalamus’s suprachiasmatic nucleus (SCN), which controls melatonin production as well as behavioral rhythms [13,16]. When that system is off balance, insomnia, trouble concentrating, mood conditions, and even far-range health conditions like heart disease and hormone-connected cancers develop [5,6,9,17,18].

While natural lighting is the ideal way of adjusting our circadian rhythms, artificial lighting is necessary for closed environments [11,12,13]. Not all artificial lighting is of equal biological significance, however. Important features of color spectrum, correlated color temperature (CCT), and luminance (lux) are instrumental in determining the impact of lighting on our sleep cycles and circadian health [13,14,15]. Office-standard fluorescent and LED lighting have different features [16,19,20]. While the old standard for many years, fluorescent lighting has been eclipsed by LED lighting because of its effectiveness and modifiable lighting quality [19,20]. Nevertheless, there is still controversy regarding what each does to our body, particularly for environments where there is no natural lighting.

Earlier research has examined the impact of artificial lighting on sleep, but not many have directly contrasted the impact of LED and fluorescent task lighting used in windowless office environments. Research has generally been conducted under tightly controlled conditions of lab tests or lighting installations different from those of normal office settings. There is an evident necessity for research conducted under realistic conditions that mimic the lighting under which people actually work.

This research aspires to fill that void by investigating 4000 K LED and 4100 K fluorescent task lighting effects on the quality of sleep and daytime performance among hospitality industry office workers who work out of a windowless environment. We seek to apply self-rated measures like the Pittsburgh Sleep Quality Index (PSQI) and daily sleep logs to determine if lighting type makes a difference for sleep and wakefulness [21,22,23]. These results contribute to the broader dialog about workplace health, lighting design, and designing facilities that encourage human wellness [5,6,17,18].

### 1.1. Significance of the Study

With an increased dependence on artificial lighting, especially in windowless rooms, there is an emerging necessity to comprehend the influence of varied lighting upon employee well-being [1,2]. Both daytime performance and the quality of sleep are crucial for maintaining general health, mental concentration, and workplace productivity. Fragmented sleep, frequent enough that it is commonly linked to out-of-place circadian rhythms, is linked to an array of health problems, including mood disturbances, heart conditions, as well as lowered cognitive processes [11,12,13,14].

While LED and fluorescent lighting are commonplace for office use, there is limited real-world data that contrasts their impact on circadian health and sleepiness under typical working conditions. This study aims to address that gap by investigating the influence of these two typical lighting sources upon subjective quality of sleep and wakefulness among office workers who work in rooms without windows [21,22,23,24].

The outcomes of this research would help guide decisions made by architects, interior designers, and facility managers regarding lighting strategies that support employee health and performance. In addition to its practical applications, the study contributes to ongoing conversations in fields such as environmental psychology, lighting ergonomics, and occupational health, where light is increasingly recognized as a key factor in human biological functioning [5,6].

### 1.2. Research Rationale and Purpose

In work environments without windows, particularly within the hospitality industry, employees often spend extended periods under artificial lighting with little to no exposure to natural daylight [1,2,3,4]. This consistent lack of natural light can disrupt the body’s circadian rhythm, leading to poor sleep quality, reduced alertness during the day, and an overall decline in well-being [11,12,13,14]. Despite lighting being a key element of workplace design, its biological and psychological impacts are frequently underappreciated in practice.

LEDs and fluorescent lamps are the most commonly used artificial lighting sources [16,19,20]. Both illuminate sufficiently for visual work but differ with respect to spectral power distribution and their impact on the human body. LEDs have gained popularity because of their high efficiency and adjustable spectrum of lighting, but there is no absolute answer as to whether they have perceivable health benefits compared to fluorescent lighting under typical office conditions, including circadian regulation and sleep, in particular [11,12,13,14]. These have mostly been conducted under lab or clinical conditions, leaving an area of missing knowledge as to what occurs under typical workplace conditions.

To address that, this research examined the effects of LED or fluorescent task lighting on perceived sleep quality and daytime functioning under windowless office conditions [16,19,20,25,26]. The results are of particular interest to employers, designers, and health professionals interested in designing more human-centered workplaces. To some extent, this work intersects between occupational health, lighting design, and chronobiology where lighting selections can potentially affect anything from productivity and cognitive performance to health outcomes in the longer term [5,6,9,17,18].

Particularly, the research assesses the impact of 4000 K LED and 4100 K fluorescent task lighting on office workers’ sleep outcomes in the hospitality industry [19,20,25,26]. Based on validated subjective measures and quasi-experimental design, the research generates evidence-based findings that underpin healthier and more productive workplace lighting strategies.

#### 1.2.1. Purpose

This research seeks to investigate if various forms of artificial task lighting, 4000 K LED and 4100 K fluorescent lamps, influence the quality of sleep and daytime functioning of office workers under windowless conditions. The particular objectives are to:

- Compare subjective sleep quality between the study’s fluorescent task-lighting source and the LED system with higher source-level melanopic content.
- Assess daytime dysfunction for each of the lighting conditions.
- Provide evidence-based recommendations for lighting design that supports circadian health for windowless rooms.

#### 1.2.2. Research Questions

Artificial lighting in windowless work environments can interfere with circadian rhythms, yet the comparative impact of commonly used light sources, such as LED and fluorescent lamps, remains insufficiently explored in real-world workplace settings. While both types of lighting meet visual performance standards, their biological effects on sleep regulation and daytime functioning may differ significantly. This study seeks to investigate whether such differences are reflected in subjective sleep quality and daily performance among office workers. To guide this inquiry, the following research questions have been formulated:

1. Does 4000 K LED task lighting have an advantage, in terms of providing better quality of subjective sleep, than 4100 K windowless office fluorescent lighting?
2. Does self-reported daytime dysfunction differ between the study’s fluorescent source and the LED system with higher source-level melanopic content?
3. How does the change in lighting conditions affect general quality of sleep scores, as rated by the Pittsburgh Sleep Quality Index (PSQI), for hospitality office employees?

#### 1.2.3. Hypotheses

Based on the current literature and known biological processes involving circadian regulation, the present study puts forth the following directional hypotheses:

**H1:**  *Participants will report significantly higher subjective sleep quality when exposed to 4000 K LED task lighting compared to 4100 K fluorescent lighting*.

**H2:**  *Daytime dysfunction scores reported by the patients will be significantly lower under LED lighting conditions than under fluorescent lighting*.

**H3:**  *Lower PSQI scores, representing improved overall sleep, will be achieved after LED task lighting exposure as compared to the use of fluorescent lighting*.

## 2. Literature Review

Human circadian rhythms are essential for controlling the sleep-wake cycle, hormone secretion, and alertness during the day [11,12]. This internal clock is mostly regulated through light, which acts through retina-located, non-visual photoreceptors to send signals to the hypothalamus-located suprachiasmatic nucleus (SCN) [13,14]. Natural daylight is the strongest signal for entraining circadian rhythms, but artificial lighting is necessary for areas where daylight is absent, including windowless rooms [13,14]. Without adequate circadian stimulation throughout the day, people can have disturbed sleep patterns, decreased alertness, and heightened risk for long-term health problems, including cardiovascular disease, depression, and metabolic disorders [14,15].

In building and workplace design, artificial lighting has conventionally been designed to address visual needs, not biological needs. Yet, spectral composition, illuminance (lux), and correlated color temperature (CCT) have a powerful impact on circadian regulation [11,15]. For instance, increased CCTs (cooler, blue-enriched lighting) have been found to boost daytime wakefulness and inhibit melatonin release, but warmer, lower-CCT lighting is more suitable for evening unwinding and bedtime preparation [13,16]. Therefore, the biological effect of light is not only determined by its quantity but also by its spectral characteristics and timing of exposure.

Circadian phase resetting commonly accrues over multiple days when bright light is delivered at biologically appropriate times, with shift magnitude strongly dependent on the phase response curve. Classic laboratory evidence shows that repeated, correctly timed bright-light exposures across several consecutive days can produce robust advances or delays of human circadian phases, far exceeding the effects of single-day stimuli. Early work demonstrated strong resetting under intense, repeated light schedules, while subsequent studies mapped the human PRC to bright light and showed that multi-day advance/delay protocols (e.g., ~3 days combined with morning bright light, sometimes paired with timed melatonin) yield cumulative phase shifts that unfold over several days [27,28,29].

The most typical lighting sources used in office contexts are fluorescent and LED lighting. Fluorescent lamps work by means of phosphor coatings powered by ultraviolet radiation, usually generating uneven spectral emissions with peaks for certain wavelengths [16,19,20]. LEDs on the other hand, have greater energy efficiency and can be designed to have a more continuum and tunable spectrum, including precise color temperature [8,19,30]. There is an indication that LED lighting of 4000–5000 K could have benefits including better visual comfort, design adaptability, and possible advantages for alertness and sleep [6,7,8,10]. Largely, however, existing research is carried out in specially designed lab contexts or for exceptional color temperature (e.g., >6500 K), which makes them limited for use on typical work contexts on a daily basis [8,19,30]. In office field settings, multi-week interventions using blue-enriched or biologically supportive spectra have reported sustained improvements in alertness, sleep, and entrainment, suggesting that exposure windows of several weeks may be necessary to observe stable changes under everyday conditions. These findings converge with broader reviews of daytime workers showing that workplace lighting can influence mood and alertness when delivered over extended periods [15,31,32].

Notwithstanding increased interest in circadian-matched design, there is still limited real-world research investigating the effect of task-level artificial lighting, especially when color temperature matched, on sleep and daytime functioning. Available field studies have difficulties segregating lighting effects because of variable control of natural lighting exposure, diverse timing of lights, and sparse use of standardized measures of sleep. Consequently, the precise LED–fluorescent distinctions, particularly under ecologically valid conditions, remain unclear [16,19,20,25,26].

Also, whereas there is broad acceptance of self-reported instruments such as the Pittsburgh Sleep Quality Index (PSQI) and sleep diaries, very few papers actually adjust for large confounding variables [18,21,22,23,24]. Variables such as individual chronotype, stress, use of caffeine, and use of electronic devices can each individually influence the endpoints of sleep, for which interpretation is problematic.

This study seeks to address these gaps by conducting a quasi-experimental investigation in a fully windowless office setting [1,2,7]. Using a within-subjects design and standardized subjective measures (PSQI and sleep diaries), the study compares 4000 K LED and 4100 K fluorescent task lighting while minimizing external light variability [16,19,20,25,26]. By isolating lighting type as the primary variable, the research aims to provide clearer insights into whether artificial light source alone, when matched in color temperature, can meaningfully affect sleep quality and daytime function in real-world office environments [21,22,23,24].

## 3. Methodology

A quasi-experimental, within-subjects crossover design was used for investigating the comparative effects of LED and fluorescent task lighting on the quality of sleep and daytime performance under realistic, windowless office setting conditions. Our purpose was to determine whether, under conditions of matched color temperature and illuminance, two of the most commonly employed artificial lighting sources have discernible subjective effects associated with sleep.

A within-subjects design was chosen to reduce inter-individual variation, and each participant acted as their own control. This type of design is most advantageous for small sample-sized investigations, as statistical power is enhanced, and confounding variables are kept to a minimum. To alleviate concerns of order effects and potential carryover, conditions of lighting exposure were displayed in a counterbalanced sequence, and participants were assigned random orders of exposure.

The experiment took place in an interior basement office that did not have natural lighting. This enclosed setting had been specially selected to reduce external lighting influences and eliminate external lighting cues. It had also been set up to have a washout between lighting conditions for the purpose of reducing residual influences and enhancing internal validity.

Both quantitative and qualitative instruments were used for collecting the data. Subjective daytime functioning and sleep quality were assessed through the Pittsburgh Sleep Quality Index (PSQI) and a daily sleep diary [21,22,23,24]. Both these instruments have good validity for use in the study of sleep, and for the purpose of the present study, the PSQI was modified to span a one-week rather than the customary one-month reporting period but otherwise remained unchanged.

Details of participant recruitment, experimental conditions, lighting requirements, procedural steps, instruments for data collection, and analytical approach employed to assess the results of the study are given under the following sections.

### 3.1. Participants

A total of 32 full-time employees (16 females, 16 males) from a hospitality service company in Colorado Springs, Colorado, participated in this study. This was a convenience sample from a single employer in the hospitality sector. While the counterbalanced within-subjects crossover design reduces between-person heterogeneity and strengthens internal validity, the single-site, single-industry sampling frame may limit external validity to broader office populations. Participants ranged in age from 24 to 56 years (M = 39.6, SD = 7.9) and held administrative, marketing, or operational positions. All individuals worked standard daytime hours (8:00 a.m. to 5:00 p.m.) in basement-level offices that lacked natural daylight, ensuring consistent lighting conditions across the sample.

Recruitment was coordinated through the company’s human resources department and carried out with executive-level approval. To be eligible, participants had to meet the following criteria: (a) no current use of sleep-related medications or supplements; (b) no diagnosed sleep disorders; (c) age 18 or older; and (d) a stable daytime schedule with no shift work or rotating hours.

Interested employees attended an orientation session where the study’s purpose, confidentiality measures, and participation requirements were explained. Informed consent was obtained from all participants prior to enrollment.

The sample size was guided by previous within-subjects studies on sleep and lighting, which suggests that a sample of 30 or more is sufficient to detect moderate effects (d = 0.50) with 80% power (β = 0.80) when using paired-samples *t*-tests. Additionally, a total of 32 participants allowed for equal distribution across the two lighting sequence orders (16 participants per sequence), ensuring balanced counterbalancing within the crossover design.

To achieve maximum experimental control, participants were requested to follow the following rules:

- Stick to steady wake-up and bedtime routines throughout the study duration.
- Refrain from consuming alcohol or caffeine for at least 24 h prior to each data collection week.
- Minimize exposure to natural light during commuting by wearing personal sunglasses; if direct sunlight remained noticeable despite sunglasses, participants were additionally instructed to lower the in-vehicle sun visor (i.e., the fold-down visor above the driver’s seat). Participants primarily used sunglasses; use of the vehicle visor was encouraged on especially bright days.
- Restrict use of screens (e.g., phones, tablets, computers) for an hour preceding bedtime.

Instructions were given in writing and supported by daily email reminders. All study procedures were also approved by the author’s Institutional Review Board (IRB# 1753587-1), and ethical human subjects research standards were scrupulously followed.

### 3.2. Lighting Conditions and Set Up

The experiment was conducted under a windowless office setting that is completely enclosed, on the basement floor of a commercial hotel building. It had no external access for natural lighting, and there were no nearby or overhead windows, skylights, or glass surfaces that might let in external lighting. In eliminating the ambient lighting interference, there was also no overhead lighting present throughout the experimental duration.

Each participant had a standardized working environment comprising an office desk, task lamp, office chair, and personal computer. The workstation monitor was an LG 24-inch IPS Full HD unit with a typical white-screen luminance of 250 cd/m^2^. This facilitated replication and consistency for all sessions. Two task lighting conditions were evaluated as the main experimental conditions and presented in Table 1.

Both lights were placed in matched architect-style swing-arm desk lamps, 20 inches (50 cm) above the desktop and angled 45 degrees to minimize direct glare. Each participant worked at a standardized office desk measuring approximately 48 × 24 inches (122 × 61 cm) with a height of about 29 inches (74 cm) and a matte light-gray laminate work surface selected to support diffuse reflection and reduce specular glare. The monitor (24-inch (61 cm) IPS, typical white-screen luminance ≈ 250 cd/m^2^) was centered at a viewing distance of ~22–26 inches (55–65 cm), with the top edge positioned ~10–15° below seated eye level to promote uniform near-field luminance and minimize veiling reflections on the display.

To limit hand-shadowing and direct reflections, the task-lamp head was positioned on the non-dominant side (left for right-hand-dominant users) and laterally offset ~10–14 inches (25–35 cm) from the monitor’s vertical centerline. The head was set ~20 inches (50 cm) above the work plane and aimed at ~30–45° incidence to illuminate the primary task area while remaining outside the monitor’s specular reflection zone (verified at the seated eye point so that the lamp was not visible in the screen’s mirror image).

**Figure 1:**
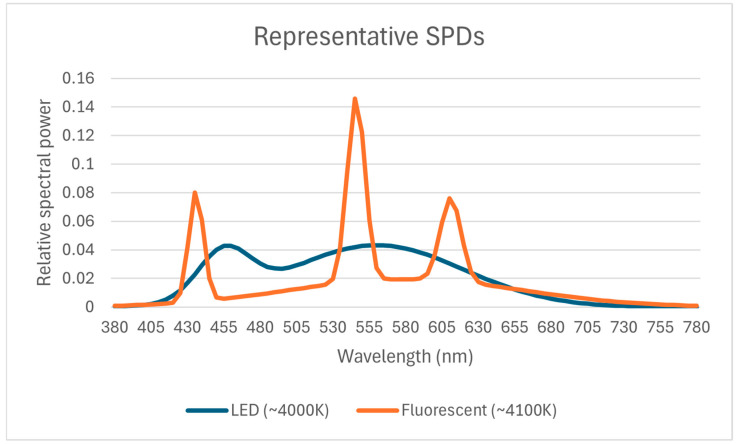
Representative spectral power distributions (SPDs) for the LED (~4000 K) and fluorescent (~4100 K) task-light sources.

Both conditions used opaque architect-style shades with white interior finish to encourage diffuse beam spread and reduce direct glare. The 4000 K LED exhibited a continuous, blue-enriched spectrum, whereas the 4100 K fluorescent showed characteristic narrow-band emission peaks; representative SPDs are provided in Figure 1. The walls and ceiling were matte white to enhance diffuse interreflections and spatial uniformity; desk-surface reflectance (LRV) was not directly measured, and the author therefore described the finish qualitatively (matte light gray). Future work will include calibrated reflectance factors and surround luminance to refine interreflection modeling for both photopic and melanopic exposure estimates.

Lamps were turned on at 8:00 a.m. and remained on until 5:00 p.m. During breaks and the lunch period, the workstation task lamp was switched off while participants were away. Participants were asked to have lunch in interior spaces without daylight whenever feasible; if they briefly went outdoors, they were instructed to wear their sunglasses and, if driving, to lower the in-vehicle sun visor.

To verify lighting consistency, the illuminance level at the center of each desk was measured using a calibrated handheld lux meter before each experimental week. Measurements were taken at five evenly spaced points across the desk surface and averaged:

- Mean illuminance under LED lighting: 405 lux (SD = 17).
- Average illuminance under fluorescent lighting: 412 lux (SD = 21).

Spectral power distributions (SPDs) for the two lighting sources are provided in Figure 1, with the LED exhibiting the typical continuous blue-enriched profile and the fluorescent source showing characteristic narrow-band spikes.

Using a calibrated spectroradiometer (Apogee MS-100, Apogee Instruments, Inc, North Logan, UT, USA), we recorded the spectral power distributions (SPDs) of both the 4000 K LED and the 4100 K fluorescent sources in situ. From these SPDs, the experimenter calculated melanopic equivalent daylight illuminance (melanopic EDI) in accordance with CIE S 026/E:2018 [33]. At the eye level at 4 feet (1.20 m) above the finished floor, the LED condition delivered ~300 lx vertical photopic illuminance (~220 melanopic lux), whereas the fluorescent condition delivered ~280 lx vertical photopic illuminance (~180 melanopic lux).

All lamps were securely mounted to prevent positional changes, multiple participants were present simultaneously in the windowless room; desks and task lamps were arranged to avoid face-to-face positioning, reducing visual distractions and cross-illumination. To further maintain environmental consistency, the room’s walls and ceiling were painted matte white to provide high diffuse reflectance and uniformity while minimizing specular glare and color cast.

Although the LED and the fluorescent lamp have only very slight color temperature difference (100 K), the present literature suggests that even slight spectral power distribution changes, particularly those of melanopic content, can have substantial circadian outcomes, also under long duration use except for natural daylight conditions [12,16].

With the 9 h daily exposure duration within an insulated, windowless room, it was anticipated that the spectral contrasts between the two lighting conditions would have biologically significant effects upon the quality of sleep as well as daytime functioning.

### 3.3. Procedure

Participants were assessed for three weeks under a within-subjects crossover design. Participants experienced each of the lighting conditions, 4000 K LED and 4100 K fluorescent task lighting, and there was also a one-week washout between exposures. To try and control order effects and potential carryover, participants were also matched to either of two counterbalanced sequences: the first half of the sample (n = 16) had the LED condition in week 1 and the fluorescent condition in week 3, and the second half had the opposite. Data collection took place in Colorado Springs, Colorado across three consecutive weeks: Week 1 (Exposure 1): 10–14 April 2023; Week 2 (Washout): 17–21 April 2023; Week 3 (Exposure 2): 24–28 April 2023.

In each exposure week, participants worked under their allocated desk settings under the prescribed task lighting condition for five contiguous weekdays (Monday to Friday). Task lamps were switched on at 8:00 a.m. and continued until 5:00 p.m., with the task lamp turned off during the lunch period while participants were away from the workstation. This is equivalent to an average full-time working day. Participants performed their typical occupation tasks, including administrative work, emails, electronic marketing campaigns, and the reading of documents. Each morning, research staff confirmed non-dominant side lamp placement, ~20 inches (50 cm) head height, and ~30–45° aiming relative to the task area and monitor to maintain the geometric conditions described above across participants and days. Tasks were primarily computer-based; however, continuous time-on-screen or gaze duration was not logged in this study.

To reduce confounding factors that might interfere with sleep quality or daytime alertness, the following behavioral controls were instituted:

- Light exposure: To minimize uncontrolled bright light exposure during commutes and the lunch break, participants were instructed to wear their own sunglasses; when sunlight was direct or intense, they were additionally asked to lower the in-vehicle fold-down sun visor. Participants were also asked to avoid brightly lit outdoor locations during lunch whenever feasible.
- Alcohol and caffeine: Participants agreed to abstain from alcohol and caffeine for 24 h or longer prior to each day of data collection.
- Sleep schedule: Participants were instructed to follow consistent bedtimes and wake times during each experimental week.
- Evening use of screens: Use of screens, such as smartphones, tablets, and computers, was avoided for one hour before bedtime to reduce suppression of melatonin due to blue light exposure.

Participants had written instructions and verbal reminders of these protocols in advance for each week of exposure, including brief midday reminders around the lunch break to remain indoors when feasible and to confirm that the task lamp was switched off while away from the workstation.

The second week of the study (Week 2) served as a washout period during which participants returned to their usual work environment without the use of any task lighting. This allowed any residual effects from the initial lighting condition to dissipate and reestablished a baseline before the second exposure.

During each week of exposure, participants filled out the daily sleep diary each morning and returned their responses to the Pittsburgh Sleep Quality Index (PSQI) on the last day of the work week (Friday). These measures were tracked and associated with the appropriate lighting condition, allowing for within-subject comparisons between the two lighting conditions.

### 3.4. Data Analysis

All statistical tests were carried out using IBM SPSS Statistics (Version 28). Given that the study design is within-subjects crossover, paired-samples *t*-tests were employed to look into participants’ subjective experiences of sleep under the LED and the fluorescent lighting conditions. This analytical method was selected due to the fact that each participant serves as his or her own control, minimizing between-person variation and maximizing statistical power.

The main dependent variables were as follows: (1) Pittsburgh Sleep Quality Index (PSQI) score, global scale, (2) component of daytime dysfunction of the PSQI, (3) subjective quality of the sleep component of the PSQI, (4) average self-reported sleep duration (from daily sleep diaries), and (5) mean self-reported quality of sleep (daily sleep diaries).

To justify the adequacy of the sample size, an a priori power analysis was conducted using a paired-samples *t*-test framework. Assuming a medium effect size (Cohen’s d = 0.50), two-tailed α = 0.05, and a desired power of 0.80, the required sample size was estimated as n ≈ 32. The final sample of n = 32 (16 females, 16 males) therefore provides sufficient statistical power to detect medium within-subjects effects. For transparency, we note that smaller effect sizes (d = 0.3–0.4) would require substantially larger samples (n ≈ 50–88) and may be underpowered in this study. Because multiple outcomes were assessed, Bonferroni correction was applied to primary comparisons to adjust for Type I error inflation.

Before conducting the *t*-tests, the assumption of normality for difference scores was assessed using the Shapiro–Wilk test and a visual inspection of Q–Q plots. Any data points exceeding 2.5 standard deviations from the mean were flagged as potential outliers for further examination but were not excluded unless justified by additional evidence.

To adjust for multiple comparisons and regulate the familywise error rate, the results were adjusted using a Bonferroni adjustment. Given that there were five main measures of outcomes, the adjusted significance threshold was *p* < 0.01 (i.e., 0.05/5).

Effect sizes for significant findings were calculated based on Cohen’s d, where conventional interpretive standards were as follows: (1) small = 0.20, (2) medium = 0.50, and (3) large = 0.80.

Ninety-five percent confidence intervals were reported for the mean differences as well as for the effect sizes for the estimation of precision around the estimates.

Descriptive statistics (mean, standard deviation, and range) for each of the outcomes under each of the lighting conditions were calculated. Preliminary tests were also carried out to examine possible order effects as well as baseline contrasts between the two counterbalancing conditions. No significant contrasts were noted at baseline, lending support to within-subject comparisons between conditions of lighting.

## 4. Results

This chapter presents the findings of the statistical comparisons of daytime functioning and sleep quality between two task lighting conditions, LED and fluorescent. Paired-samples *t*-tests, based on a within-subjects crossover design, were conducted to compare differences between the PSQI and daily sleep diary-derived measures of outcome. For context, spectral measurements indicated higher melanopic stimulus under the LED condition (vertical melanopic EDI ≈ 220 lx) than the fluorescent condition (≈180 lx) at comparable vertical photopic illuminances (~300 vs. ~280 lx). These physiological light metrics align with the observed improvements in the PSQI global and component scores under LED lighting.

Variables were chosen to represent overall as well as component-level effects of lighting conditions on sleep outcomes.

Results are reported below (Table 2), starting with overall PSQI scores, followed by subcomponent analysis and diary measures. For each measure, descriptive statistics, significance values, effect sizes (Cohen’s d), and 95% confidence intervals are listed to facilitate interpretation and determine the practical significance of the noted effects.

### 4.1. PSQI Global Scores

Participants exhibited a statistically significant reduction in the global Pittsburgh Sleep Quality Index (PSQI) score. The mean global PSQI score under LED lighting was 4.53 (SD = 1.47), while the mean score under fluorescent lighting was higher at 5.44 (SD = 1.71), indicating poorer sleep quality.

A paired-samples *t*-test confirmed that the difference here also achieved statistical significance, *t*(31) = –3.08, *p* = 0.004. Based on Cohen’s d, the effect size calculated equaled 0.55, also reflective of a moderate effect for the LED condition. The 95% confidence interval for the mean difference (–1.52 to –0.31) indicates a reliable within-subject benefit for the LED condition in this sample. Although statistically significant (*t*(31) = –3.08, *p* = 0.004; Cohen’s d = 0.55), the mean PSQI under LED remained near the threshold value of five, indicating a modest improvement rather than a clinically superior sleep profile.

These findings suggest that LED task lighting is associated with modest improvements in PSQI global scores compared with typical windowless office fluorescent lighting.

### 4.2. PSQI Components

To better understand the particular features of sleep quality that are affected by lighting conditions, two of the most prominent subcomponents of the Pittsburgh Sleep Quality Index (PSQI) were examined individually: subjective quality of sleep and daytime dysfunction. Focusing on these subcomponents individually permitted better insight into the way that task lighting might differentially affect participants’ views of sleep and their daytime functioning.

#### 4.2.1. Subjective Sleep Quality

They also rated their subjective quality of sleep as being significantly better under LED lighting conditions than under fluorescent conditions. Mean quality of sleep under LED conditions was 0.91 (SD = 0.50), but under the fluorescent lighting, mean quality of sleep was 1.28 (SD = 0.61).

A paired-samples *t*-test found this difference to be statistically significant, *t*(31) = −3.27, *p* = 0.002. Cohen’s d, as an estimate of the effect size, was 0.58, which is suggestive of a moderate to large effect in favor of the LED condition. The 95% confidence interval for the mean difference was [−0.59, −0.15], providing supporting evidence of a steady enhancement of perceived sleep quality under LED lighting.

#### 4.2.2. Daytime Dysfunction

Participants also reported that they had less difficulty performing throughout the daytime following LED lighting presentation (M = 0.75, SD = 0.58) as compared to after the presentation of fluorescent lighting (M = 1.06, SD = 0.61) as shown in Table 3. A paired-samples *t*-test confirmed that the difference between these means was, indeed, statistically significant, *t*(31) = −2.75, *p* = 0.010 (Table 3). Effect size, calculated based on Cohen’s d, equaled 0.49, indicating an apparent moderate positive LED lighting effect on daytime alertness and functioning. The 95% confidence interval for the difference between means was [−0.55, −0.07].

These findings indicate that benefits of LED lighting went beyond better nocturnal sleep to better daytime functioning, also providing further support for the proposition that lighting that is supportive of the circadian rhythm can have multidimensional benefits, even for windowless office rooms.

### 4.3. Daily Sleep Diary Outcomes

In addition to the weekly PSQI assessments, participants completed daily sleep diaries for each morning of the LED and fluorescent exposure weeks. Two primary variables were extracted from these diaries: (1) mean self-reported length of sleep (in hours), and (2) mean self-rated quality of sleep, rated on a 5-point Likert scale (1 = very poor to 5 = very good). These daily measures permitted an additional perspective on the PSQI scores, allowing for more detailed, in-real-time observations of the effect of lighting conditions on participants’ nightly experience of sleep.

#### 4.3.1. Sleep Duration

Participants reported slightly longer average sleep durations during the LED lighting condition (M = 6.97 h, SD = 0.73) compared to the fluorescent condition (M = 6.81 h, SD = 0.68). A paired-samples *t*-test indicated that this difference approached significance, *t*(31) = 1.97, *p* = 0.058, but did not reach the adjusted significance threshold following Bonferroni correction (*p* < 0.01). The effect size, as measured by Cohen’s d, was 0.35, suggesting a small to moderate effect in favor of LED lighting. The 95% confidence interval for the mean difference was [−0.01, 0.33]. Even though it is not significant, this tendency suggests a possibly positive impact of LED lighting exposure on time asleep, also needing follow-up evaluation on bigger sample or longer duration of exposure-based studies.

#### 4.3.2. Self-Rated Sleep Quality

On a 5-point Likert scale ranging from 1 (very poor) to 5 (very good), participants rated their sleep quality significantly higher during the LED lighting condition (M = 3.63, SD = 0.68) compared to the fluorescent condition (M = 3.28, SD = 0.71). A paired-samples *t*-test confirmed this difference was statistically significant, *t*(31) = 2.96, *p* = 0.006. The effect size, calculated using Cohen’s d, was 0.52, indicating a moderate effect in favor of the LED condition. The 95% confidence interval for the mean difference was [0.11, 0.60], reflecting a reliable improvement in perceived sleep quality under LED lighting.

Both of these diary-based findings correspond to the PSQI results and indicate that LED task lighting could have subtle but steady advantages for sleep quality even for comparatively short exposure times under very tightly regulated, windowless office conditions.

### 4.4. Order Effects

To determine whether the sequence of lighting exposure influenced the study outcomes, preliminary analyses were conducted comparing the two counterbalanced groups:

- Group A: LED lighting during Week 1, Fluorescent lighting during Week 3.
- Group B: Fluorescent lighting during Week 1, LED lighting during Week 3.

Independent-samples *t*-tests were performed on the baseline (Week 1) global PSQI scores, as well as on the Week 3 PSQI scores, to identify any differences between the two groups. Results showed no statistically significant differences at either time point (*p*s > 0.10), suggesting that baseline sleep quality and end-of-study outcomes were not influenced by exposure order.

More detailed analysis of the measures from the daily sleep diary did not reveal substantial presentation order by lighting condition interactions. These results strengthened the power of the within-subjects crossover design and confirmed that the difference between LED and fluorescent lighting conditions that had been identified did not arise due to the condition sequence that participants had been exposed to.

## 5. Discussion

This study examined the differential effects of LED versus fluorescent task lighting on sleep quality and daytime functioning in a windowless office environment. Consistent with the study’s hypotheses, the LED condition yielded statistically lower PSQI global scores and higher diary-based sleep-quality ratings than the fluorescent condition.

Findings were consistent across both weekly (PSQI) and daily (sleep diary) measures. The PSQI data indicated significant improvements in global sleep quality, subjective sleep quality, and daytime functioning under the LED condition. Complementing these results, daily sleep diaries showed higher self-rated sleep quality and a marginal trend toward increased sleep duration when participants were exposed to LED lighting. The convergence of findings across multiple time scales and instruments strengthens the reliability of the results and suggests that LED lighting may not only improve sleep-related outcomes but also enhance daytime cognitive functioning and alertness.

Beyond nominal CCT matching, our in situ SPD recordings and CIE S 026/E:2018 [28] aligned melanopic EDI calculations demonstrated a meaningful melanopic advantage for the LED condition (eye-level melanopic EDI ≈ 220 lx) relative to the fluorescent condition (≈180 lx) at comparable vertical photopic illuminances (~300 vs. ~280 lx). Consistent with typical 4000 K white-LED spectra, the LED source provided greater melanopic content in the ipRGC-sensitive region, which plausibly engaged non-visual pathways and aligns with the lower PSQI global scores and improved diary-based sleep outcomes observed under LED in this study.

The findings carry practical implications for lighting design in office environments, particularly those without access to natural daylight. Selecting task lighting that incorporates biologically supportive spectral characteristics, such as continuous-spectrum LEDs with moderate blue content, may serve as a cost-effective strategy to promote employee health and performance. These design choices could be integrated into broader ergonomic and biophilic frameworks that prioritize circadian-friendly indoor environments.

However, several limitations must also be mentioned. Firstly, although the study sample had been increased to 32 participants, equally divided between the sexes, the sample is still rather modest, putting an upper limit on power to detect subtle effects or to conduct subgroup analysis (e.g., by chronotype or across several subgroups of different ages). Secondly, all outcomes were self-reported, risking bias or subjective variability. Objective physiological measures (e.g., actigraphy, salivary melatonin or cortisol, polysomnography) should be included in future work to confirm and generalize these findings. Thirdly, the short duration of exposure, being limited to just two one-week regimes of lighting, prevented an exploration of longer-term effects, including adaptation or of the cumulative benefits of circadian-compatible lighting with time. Fourth, although the experimenter instructed participants to wear sunglasses and, as needed, to lower the vehicle sun visor during commutes, we did not standardize or record sunglasses characteristics (e.g., visible or melanopic transmission, spectral filtering, wrap geometry), which introduces unquantified variability in pre-work light exposure. Fifth, while we quantified SPDs, vertical photopic illuminance, melanopic EDI, and maintained wall luminance within a narrow range (~30–35 cd/m^2^), we did not directly measure wall/ceiling light reflectance values (LRVs). Future work should obtain calibrated reflectance factors to model interreflections and refine predictions of both photopic and melanopic exposures at the eye. Additionally, because participants’ tasking was predominantly computer-based, we did not log continuous time focusing on the computer screen or gaze behavior during work hours. Personal eye-level light dose during tasking therefore remained unquantified and should be captured via wearable light logging and/or eye-level vertical illuminance measurements in future studies.

Because participants were recruited from one employer in a single industry and participation was voluntary, selection effects cannot be ruled out. The demographic and occupational composition of the cohort may differ from other office sectors and from shift work or field/creative roles. Accordingly, transportability is most defensible to comparable, dayshift, windowless office settings with similar eye-level vertical photopic (~300 lx) and melanopic EDI (~180–220 lx) exposures. Results cannot be assumed to generalize to shift workers, settings with substantial daylight exposure, physically demanding or safety-critical tasks, or populations with different age or chronotype distributions. Future research should implement multi-site, multi-industry designs with stratified recruitment (age, gender, chronotype, job type), extend follow-up, and include objective light-dose logging to test industry–lighting interactions. Additionally, a priori power analysis confirmed that the sample size of n = 32 was sufficient to detect medium within-subjects effects (d = 0.50) at 80% power and α = 0.05. However, the study may be underpowered to detect smaller effects, which should be addressed in future research with larger samples.

In spite of these restrictions, the present study presents useful proof that LED task lighting, when properly specified for spectral quality, can have beneficial effects on nocturnal sleep as well as daytime performance under artificial lighting conditions. These findings are an additional contribution to the body of literature that argues for light-informed, health-centered design directions for buildings as well as workspaces. Adding circadian-friendly lighting features into workspaces could be an effective and scalable intervention for promoting employee health as well as productivity within today’s increasingly built-in workplace environments.

## 6. Conclusions

This experiment examined LED and fluorescent task lighting impacts on sleep quality and daytime functioning under windowless office conditions. It employed a within-subject cross-over design involving 32 participants. Findings indicated that the LED system used in this study was associated with a statistically significant reduction in PSQI global scores (mean difference ≈ 0.91 points; Cohen’s d = 0.55) and with higher diary-based sleep-quality ratings, alongside lower daytime dysfunction relative to the fluorescent source used in this study.

The results add to the body of literature underscoring the significance of light quality, particularly spectral composition, for promoting circadian health. For environments where access to natural daylight is restricted or completely absent, the adoption of continuous-spectrum, blue-enriched LED lighting could be an easy but effective means of yielding benefits for nocturnal sleep as well as for daytime cognitive functioning.

Specifically, multi-site, multi-industry studies with stratified sampling and objective light-dose monitoring are needed to confirm transportability beyond hospitality office settings. Although results are promising, additional research is required to generalize these outcomes for longer intervals of time and more diverse populations. Future studies should involve objective measures of physiology (e.g., actigraphy, melatonin levels) and examine the way that individual characteristics (e.g., chronotype, age, work obligations) moderate lighting’s impact on alertness and sleep.

Nevertheless, the present study underscores the practical value of circadian-aware lighting interventions. Thoughtful selection of task lighting, especially in enclosed or subterranean office settings, may represent a cost-effective and scalable approach to promoting employee well-being, productivity, and long-term health.

## Figures and Tables

**Table 1:** Lighting Conditions and Experimental Schedule.

Condition	Week	Light Type	Correlated Color Temperature (CCT)	SPD Type	Peak Wavelength	Horizontal Illuminance at Desk (lx)	Daily Exposure Duration
Condition A	1	LED	4000 K	Continuous-spectrum	~460 nm (blue peak)	405 ± 17 lx	8:00 a.m.–5:00 p.m.
Condition B	3	Fluorescent	4100 K	Narrow-band spiked SPD	~550 nm (green peak)	412 ± 21 lx	8:00 a.m.–5:00 p.m.

Horizontal illuminance values reflect five-point desk-plane measured averages under the geometry described in Methods (lamp head ≈ 20 inches (50 cm) above the work plane, ~30–45° aiming, ~10–14 inches (25–35 cm) lateral offset from the monitor centerline).

**Table 2:** Descriptive statistics for sleep outcomes under LED and fluorescent conditions.

Outcome Variable	LED (M ± SD)	LED 95% CIs	Fluorescent (M ± SD)	Fluorescent 95% CIs
PSQI Global Score	4.53 ± 1.47	[4.00, 5.06]	5.44 ± 1.71	[4.82, 6.06]
PSQI: Subjective Sleep Quality	0.91 ± 0.50	[4.00, 5.06]	1.28 ± 0.61	[1.06, 1.50]
PSQI: Daytime Dysfunction	0.75 ± 0.58	[0.54, 0.96]	1.06 ± 0.61	[0.84, 1.28]
Sleep Duration (h)	6.97 ± 0.73	[6.71, 7.23]	6.81 ± 0.68	[6.56, 7.06]
Sleep Quality Rating (1–5)	3.63 ± 0.68	[3.38, 3.88]	3.28 ± 0.71	[3.02, 3.54]

**Table 3:** Descriptive statistics and group comparisons for PSQI components.

PSQI Component	LED (M ± SD)	LED 95% CIs	Fluorescent (M ± SD)	Fluorescent 95% CIs	*t*(31)	*p*	Cohen’s d
Subjective Sleep Quality	0.91 ± 0.50	[0.73, 1.09]	1.28 ± 0.61	[1.06, 1.50]	−3.27	0.002	0.55
Sleep Latency	1.13 ± 0.64	[0.90, 1.36]	1.16 ± 0.67	[0.92, 1.40]	−0.25	0.804	0.58
Sleep Duration	0.91 ± 0.53	[0.72, 1.10]	0.97 ± 0.57	[0.76, 1.18]	−0.63	0.534	0.49
Habitual Sleep Efficiency	0.75 ± 0.62	[0.53, 0.97]	0.84 ± 0.67	[0.60, 1.08]	−0.68	0.501	0.35
Sleep Disturbances	1.28 ± 0.44	[1.12, 1.44]	1.38 ± 0.49	[1.20, 1.56]	−0.93	0.359	0.52
Use of Sleep Medication	0.00 ± 0.00	[0.00, 0.00]	0.00 ± 0.00	[0.00, 0.00]	-	-	-
Daytime Dysfunction	0.75 ± 0.58	[0.54, 0.96]	1.06 ± 0.61	[0.84, 1.28]	−2.75	0.010	

## Data Availability

The data presented in this study are available on request from the corresponding author due to privacy.

## Abbreviations

The following abbreviations are used in this manuscript:

CRI	Color Rendering Index
K	Kelvin
L	Lumens
LED	Light Emitting Diode
